# S‐9‐PAHSA's neuroprotective effect mediated by CAIII suppresses apoptosis and oxidative stress in a mouse model of type 2 diabetes

**DOI:** 10.1111/cns.14594

**Published:** 2024-02-08

**Authors:** Jian‐tao Wang, Xin‐ru Wang, Jiao‐qi Ren, Jin‐hong Lin, Zhong‐yu Yu, Shan‐shan Huang, Yue Hu, Jia‐yu Fu, Meng Wang, Yan‐li Zhang, Xue‐chun Wang, Jing‐chun Guo, Ji‐chang Xiao, Hou‐guang Zhou

**Affiliations:** ^1^ Department of Geriatric Neurology of Huashan Hospital, National Clinical Research Center for Aging and Medicine Fudan University Shanghai China; ^2^ Department of General Practice Affiliated Hospital of Xuzhou Medical University Xuzhou Jiangsu Province China; ^3^ Key Laboratory of Organofluorine Chemistry, Shanghai Institute of Organic Chemistry University of Chinese Academy of Sciences, Chinese Academy of Sciences Shanghai China; ^4^ State Key Laboratory of Medical Neurobiology, MOE Frontier Center for Brain Science, Department of Translational Neuroscience of Shanghai Jing'an District Centre Hospital Institutes of Brain Science, Fudan University Shanghai China

**Keywords:** carbonic anhydrase III, mitochondrial function, oxidative stress, S‐9‐PAHSA, type 2 diabetes mellitus

## Abstract

**Background:**

With the rapidly increasing prevalence of metabolic diseases such as type 2 diabetes mellitus (T2DM), neuronal complications associated with these diseases have resulted in significant burdens on healthcare systems. Meanwhile, effective therapies have remained insufficient. A novel fatty acid called S‐9‐PAHSA has been reported to provide metabolic benefits in T2DM by regulating glucose metabolism. However, whether S‐9‐PAHSA has a neuroprotective effect in mouse models of T2DM remains unclear.

**Methods:**

This in vivo study in mice fed a high‐fat diet (HFD) for 5 months used fasting blood glucose, glucose tolerance, and insulin tolerance tests to examine the effect of S‐9‐PAHSA on glucose metabolism. The Morris water maze test was also used to assess the impact of S‐9‐PAHSA on cognition in the mice, while the neuroprotective effect of S‐9‐PAHSA was evaluated by measuring the expression of proteins related to apoptosis and oxidative stress. In addition, an in vitro study in PC12 cells assessed apoptosis, oxidative stress, and mitochondrial membrane potential with or without CAIII knockdown to determine the role of CAIII in the neuroprotective effect of S‐9‐PAHSA.

**Results:**

S‐9‐PAHSA reduced fasting blood glucose levels significantly, increased insulin sensitivity in the HFD mice and also suppressed apoptosis and oxidative stress in the cortex of the mice and PC12 cells in a diabetic setting. By suppressing oxidative stress and apoptosis, S‐9‐PAHSA protected both neuronal cells and microvascular endothelial cells in in vivo and in vitro diabetic environments. Interestingly, this protective effect of S‐9‐PAHSA was reduced significantly when CAIII was knocked down in the PC12 cells, suggesting that CAIII has a major role in the neuroprotective effect of S‐9‐PAHSA. However, overexpression of CAIII did not significantly enhance the protective effect of S‐9‐PAHSA.

**Conclusion:**

S‐9‐PAHSA mediated by CAIII has the potential to exert a neuroprotective effect by suppressing apoptosis and oxidative stress in neuronal cells exposed to diabetic conditions. Furthermore, S‐9‐PAHSA has the capability to reduce fasting blood glucose and LDL levels and enhance insulin sensitivity in mice fed with HFD.

## Graphical abstract

S‐9‐PAHSA has the potential to exert neuroprotective effects mediated by CAIII via suppressing apoptosis and oxidative stress in neuronal cells exposed to diabetic conditions. Furthermore, it has the capability to reduce fasting blood glucose and LDL and enhance insulin sensitivity in mice with a high‐fat diet.

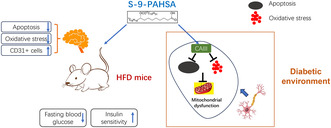

## 1 BACKGROUND

With the rapid increase in population aging and the gradual transition of changes in the daily diet, the prevalence of metabolic diseases such as type 2 diabetes mellitus (T2DM) and hyperlipidemia has increased in elderly people. This situation is particularly evident in China, a novel emerging developing country. A recent cross‐sectional study in 31 provinces in China reported that the prevalence of T2DM in adults in 2018 was 11.2%.^1^ Another study in China also reported a prevalence of approximately 23.3% in individuals aged 60–69 years and 26.6% in those aged over 70 years, with the prevalence of metabolic diseases in these elderly people also high, and poorly controlled.^2^ Common complications of disorders related to glucolipid metabolic diseases, such as T2DM, include cardiovascular disease, renal disorders, heart failure, atherosclerosis, and cognitive impairment. These complications impose heavy burdens on the physical and mental health of the elderly population.^3^, ^4^, ^5^ Insulin resistance is more closely linked to T2DM compared with that of islet β cell dysfunction, with this trend more pronounced in the obese population. These findings suggest that insulin resistance is a potential target for intervention in the management of T2DM.^6^

Oxidative stress is a pathological condition caused by imbalance between the anti‐oxidative stress system and the production of reactive oxygen species (ROS) and reactive nitrogen species (RNS). Several studies have shown that oxidative stress is one of the most important pathological changes in neuronal and vascular lesions related to T2DM and that suppression of key proteins in oxidative stress pathways contributes to a reduction in the development of insulin resistance.^7^, ^8^, ^9^ However, limited progress has been made in developing effective interventions that target oxidative stress in neurovascular complications related to T2DM.^10^, ^11^ Vascular lesions are also one of the main complications of T2DM,^12^ with atherosclerosis being the most significant pathological feature. There is also evidence that oxidative stress is associated closely with the development of atherosclerosis,^13^ with suppression of NADPH oxidase alleviating this pathological change.^11^ In view of the important role of oxidative stress in T2DM and its neurological complications, it has become an ideal intervention to prevent these complications. In addition, apoptosis is a typical pathological feature of T2DM and is therefore also a therapeutic target for preventing neurovascular complications associated with the disorder.^14^, ^15^

Palmitic acid hydroxy stearic acid (PAHSA) has emerged as a novel form of palmitic acid and has become a focal point in studies on metabolic disorders. Previous studies have shown that PAHSA improves insulin sensitivity in hepatocytes and protects islet β cells under diabetic conditions.^16^, ^17^ The 9‐PAHSA isomer of the fatty acid has been shown to activate the G‐protein coupled receptor 40 (GPR40), thereby enhancing insulin sensitivity and alleviating chronic inflammation in adipose tissue,^18^ in addition to protecting hepatocytes from steatosis by maintaining mitochondrial function.^19^, ^20^ Our previous study in DB/DB mice demonstrated that 9‐PAHSA also improved cardiac functions by upregulating autophagy flux and enhancing cognitive function.^21^ However, the underlying mechanisms of these actions remain unclear, indicating the need for further studies in this area. Recent studies have shown that 9‐PAHSA has two distinct stereoconfigurations, R‐9‐PAHSA and S‐9‐PAHSA, with evidence that the S‐9 configuration stimulates insulin secretion and glucose uptake to a greater extent than that of the R‐9 configuration.^22^

Carbonic anhydrases (CAs) are metalloproteinases known for their ability to catalyze the conversion of CO_2_. Of the 15 subtypes of CAs, CAIII exhibits low catalytic activity, with its antioxidant effects having attracted significant attention in recent years.^23^ Two types of cysteine residues, Cys183 and Cys188, located on the surface of CAIII, have been shown to have a potential antioxidant function by undergoing S‐glutathionylation.^24^ Recent studies have reported that CAIII has an antioxidant role in skeletal muscles, with its downregulation in disc nucleus pulposus cells demonstrated to exacerbate injuries under enhanced oxidant conditions.^23^, ^25^ Other studies have also shown that the activity of CAs is associated closely with brain function and cognition, with agonists of CAs demonstrated to enhance cognitive function.^26^, ^27^, ^28^ These results indicate that CAIII may play a role in normal cognition and anti‐oxidant activity. Our previous study demonstrated that CAIII was downregulated in the myocardium and cortex in mouse models of T2DM, suggesting its involvement in the pathological progression of the disorder and its complications.^29^ However, the exact molecular mechanism of these effects still requires further investigation.

Taken together, these findings indicate that oxidative stress is involved in the neuronal pathology of T2DM and that S‐9‐PAHSA may play a key role in neuroprotection as well as influencing glucolipid metabolism.^28^, ^29^ Considering the similar biological activity of CAIII and PAHSA on anti‐oxidative stress, and cognitive function in T2DM, it is possible that CAIII may be involved in the protective effect of S‐9‐PAHSA. However, evidence to confirm this relationship and its specific mechanisms requires further study.

## 2 METHODS AND MATERIALS

### 2.1 Synthesis of PAHSA

In collaboration with Professor Jichang Xiao's team from the Shanghai Institute of Organic Chemistry, Chinese Academy of Sciences, we independently synthesized 5‐PAHSA and 9‐PAHSA using the method reported in 2014 by Yore et al.^30^ Following synthesis, we conducted purity identification tests, which confirmed that the purity of the compounds met our expectations. With the successful synthesis of 5‐PAHSA and 9‐PAHSA, we proceeded to investigate a new pathway for the independent synthesis of S‐9‐PAHSA, which is described in detail in Appendix S1 and S2 along with the results confirming purity.

### 2.2 Animals and grouping

Male C57BL/6J mice were purchased from Jiangsu Jicui Yaokang Biotechnology Company. The mice were kept in a room at a constant temperature and humidity, with light alternated between light and dark for 12/12 h. Mice with T2DM induced by a high‐fat diet (HFD) are a classic mouse model of glucose and lipid metabolism disorders^31^ and were therefore chosen as a suitable animal model for the purposes and aims of our study.

**FIGURE 1:**
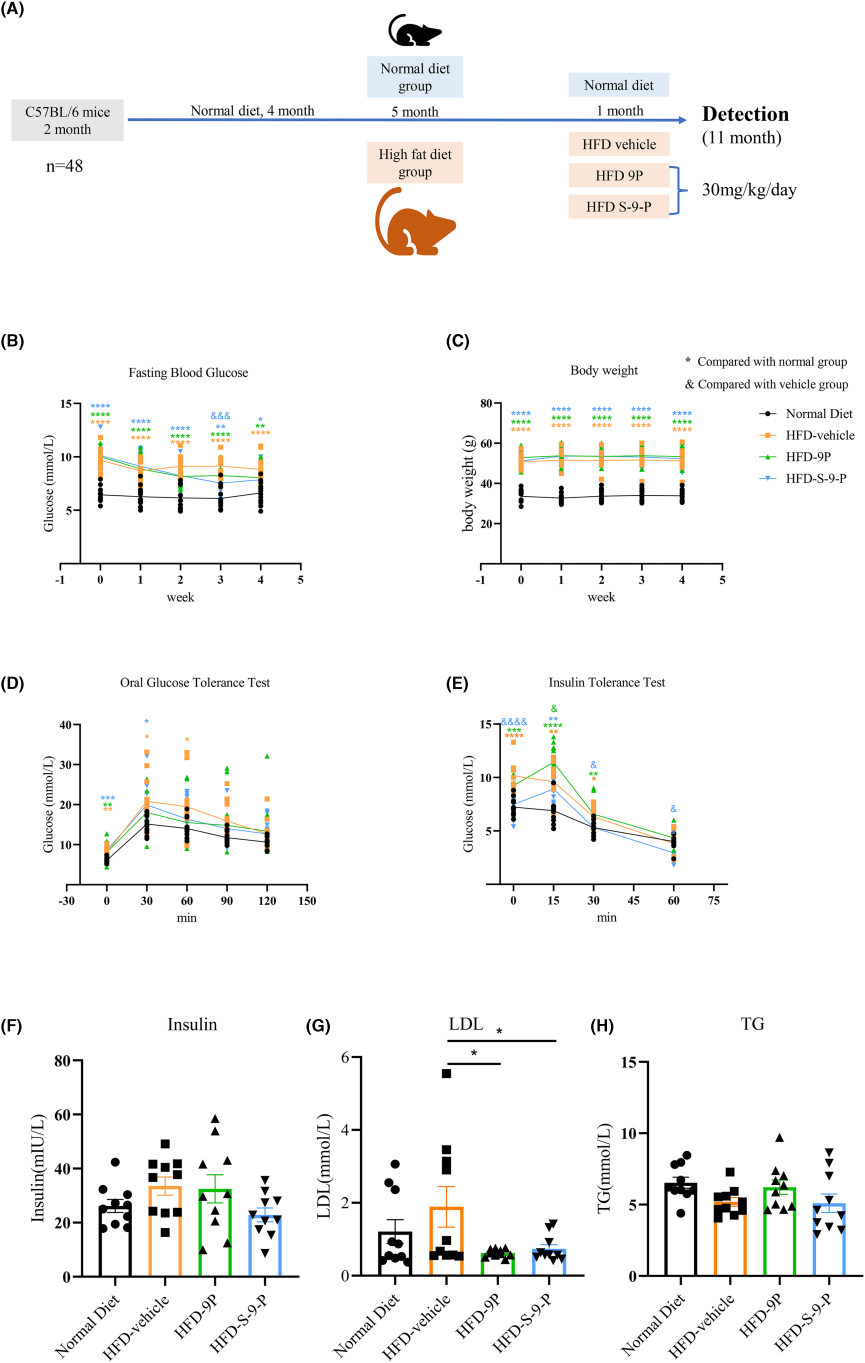
Glucolipid metabolism of mice who received different administrations for 4 weeks. (A) Animal grouping and PAHSA administration. (B and C) Fasting blood glucose levels and body weight of mice in the different groups. (D and E) Results of the OGTT and ITT in the mice in different groups. * vs. normal groups, ^&^ vs. vehicle groups, *p < 0.05, **p < 0.01, ***p < 0.001, ****p < 0.0001, ^&^ *p* < 0.05, ^&&^ *p* < 0.01, ^&&&^ *p* < 0.001, ^&&&&^ *p* < 0.0001. (F–H) Serum insulin, LDL and TG levels in the different mice. *p < 0.05 vs. vehicle groups, *n* = 10–12. The data are shown as mean ± SEM. One‐way ANOVA was used in B–H.

A total of 48 C57BL/6J mice were fed a normal diet until 6 months old and then selected randomly to receive a normal diet (ND, *n* = 12) or an HFD containing 60% fat (*n* = 36). One week before receiving the official HFD, the HFD group was fed a diet containing high fat and a normal mixture in order for them to adapt to the HFD. After this 1‐week adaptation, the HFD was added and continued for 5 months. The procedures used for grouping and PAHSA administration are shown in Figure 1A. All the animal‐related experiments were approved by the Experimental Animal Ethics Committee of Shanghai Medical College, Fudan University (2020‐Huashan hospital‐JS190).

### 2.3 Administration of PAHSA to the mice

In accordance with previous studies, 9‐PAHSA and S‐9‐PAHSA were diluted in a vehicle that included 49.5% polyethylene glycol‐400 (PEG‐400), 0.5% Tween‐80, and 50% H_2_O.^30^ The HFD mice were divided into three groups to receive either vehicle alone, 9‐PAHSA, or S‐9‐PAHSA at a dosage of 30 mg/kg/day orally for 28 days.

### 2.4 Measurement of fasting blood glucose and the methodology of the insulin tolerance and oral glucose tolerance tests

Measurement of fasting blood glucose levels in the mice was conducted every 7 days at 9:00 am after the animals had fasted for 14 h. The mice were placed gently on a plate and a sterile scalpel blade was used to gently cut the tail vein to obtain a blood sample for measurement of blood glucose levels. An insulin tolerance test (ITT) and oral glucose tolerance test (OGTT) were then performed after administration of S‐9‐PAHSA. The ITT was conducted at 3:00 pm (GMT+8), the mice fasted for 3 h, followed by the measurement of their blood glucose level. Insulin was then injected intraperitoneally (1 U/kg) and blood glucose levels were measured after 15, 30, and 60 min. The OGTT was performed 1 day after the ITT at 9:00 am (GMT + 8), with the mice fasted for 14 h, followed by measurement of their blood glucose level. Glucose (1 mg/kg) was then administered by gavage and the blood glucose levels were measured after 30, 60, 90, and 120 min, the mice were sacrificed 1 day after ITT and OGTT.

### 2.5 Behavior test of the mice

The Morris water maze test was conducted 1 day after S‐9‐PAHSA administration and 7 days before the ITT and OGTT to assess cognition in the mice. The mice were placed into a container of water and allowed to freely explore the environment. The container was divided equally into four quadrants, with the platform placed randomly in a quadrant and submerged about 2 cm under the water. White food additives were added to the container to prevent the mice from seeing the platform. The test consisted of two parts; the positioning navigation experiment and the space exploration experiment. In the positioning navigation experiment, the first 5 days were the training period, with the mice placed in four different quadrants at a fixed time every day. The period of time the mice took from entering the water to finding the platform was recorded as the latency. If a mouse could not find the platform within 60 s, they were guided to the platform and the latency was recorded as 60 s. All the mice rested for 10 s after boarding the platform. After 5 days of training, the positioning navigation test was repeated on the sixth day, with latency measured using the same method. The space exploration experiment was carried out after the navigation experiment by removing the platform and placing the mice in the opposite quadrant to where the platform was located. The swimming time, distance, speed in the target quadrant, and the time the mouse took to cross to the platform was then recorded.

### 2.6 Western blotting and ELISA

Western blotting were performed to detect the expression of proteins according to the detailed procedure described previously.^32^ The primary antibodies were purchased from Cell Signal Technology (Boston, USA) and included β‐actin, Bcl‐2, Bax, Bcl‐xl, cleaved caspase‐3, p‐Bad (Ser112), Txn2, SOD2, and HO‐1. gp91‐phox was purchased from Santa Cruz (Dallas, USA). With the exception of β‐actin which was diluted at 1:10,000, all the other antibodies were diluted at 1:2000. The ELISA's for insulin, LDL cholesterol, and triglyceride (TG) was conducted according to the manufacturer's instructions (DiLab, Shanghai, China).

### 2.7 Immunofluorescence

The mice were infused with 4% paraformaldehyde before sacrifice, with the brain fixed for 24 h in a 4°C freezer, followed by dehydration in 30% sucrose solution. The brain was embedded in an optical cutting temperature (OCT) medium and sliced after freezing. The sections were rinsed three times with phosphate buffer saline (PBS), blocked by bovine serum albumin (BSA) for 30 min, and the appropriate proportions of CD31 primary antibody were added according to the manufacturer's instructions. The sections were then incubated overnight at 4°C in the refrigerator, rinsed three times in PBS, and the corresponding species of secondary antibody added. Following incubation for 50 min in the dark and a further three rinses in PBS, the sections were incubated with the secondary antibody, rinsed in PBS three times, and then incubated with DAPI stain solution for 10 min. After sealing with a mounting agent the sections were observed using a fluorescence microscope.

### 2.8 Cell culture

A highly differentiated rat adrenal pheochromocytoma cell line (PC12) was purchased from Fuheng Biotechnology (Shanghai, China), and rat brain vascular endothelial cells (RBE4) from Yaji Biotechnology (Shanghai, China). The PC12 and RBE4 cells were cultured in an incubator (37°C, 5% CO_2_) with RPMI‐1640 medium and DMEM containing 10% fetal bovine serum (FBS) and 1% penicillin/streptomycin, respectively.

### 2.9 Overexpression or knockdown of CAIII in PC12 cells

Lentiviral vectors targeting CAIII sequence (shCAIII) and the short hairpin RNA were designed and synthesized from RiboBio (Shanghai, China). The sequences were as follows: shCAIII: 5′‐GCATGAACTTTATCCAATTGC‐3′, NC: 5′‐TTCTCCGAACGTGTCACGT‐3′. Subsequently, the lentivirus housing shRNA or NC was generated via co‐transfection in 293 T cells. PC12 cells were seeded into 12‐well plates. After overnight incubation, the original culture medium was aspirated, and the medium containing lentiviral liquid was added. Then, after 16 h of infection, the culture medium containing lentivirus was replaced with a normal culture medium. Two days later, the infection efficiency was detected by a fluorescence microscope (Olympus, Japan). The overexpression vectors targeting CAIII (gene ID: 54232, NM_019292.4) were also constructed to establish the CAIII overexpressed PC12 cells.

### 2.10 Establishment of the in vitro cell T2DM model

Adding additional glucose to a cell culture medium is a common way to prepare an in vitro model of T2DM. According to the literature, the glucose concentration in PC12 cells may vary when used as an in vitro model of T2DM.^33^, ^34^ Adding free fatty acids (FFAs) to a cell culture medium is also a common way to construct an in vitro hyperlipidemia model.^35^, ^36^ Therefore, we chose glucose plus FFA to construct an in vitro cell model of a glucose and lipid metabolism disorder. Based on reports in the literature and previous experimental results, we showed that for PC12 cells, a glucose concentration of 100 mM and an FFA concentration of 200 μM was the lowest concentration that significantly inhibited cell proliferation. The corresponding lowest concentration for RBE4 cells was a glucose concentration of 100 mM and an FFA concentration of 100 μM. Therefore, the model of the PC12 cell glucose and lipid metabolism disorder contained 100 mM glucose and 200 μM FFA (hereinafter referred to as G100F200), while the model for the RBE4 cell glucose and lipid metabolism disorder contained 100 mM glucose and 100 μM FFA (hereinafter referred to as G100F100).

### 2.11 ROS detection

The cells were seeded in 24‐well plates and after being treated with 9‐PAHSA or S‐9‐PAHSA (60 μM) for 24 h, the medium was removed and the cells were washed twice with PBS, followed by the addition of DCFH‐DA (Dojindo Laboratories, Japan) and incubation for 30 min in the dark at 37°C with 5% CO_2_. After culturing, the cells were washed three times with a serum‐free medium, followed by the addition of fresh medium and detection of the ROS level using a fluorescent microscope. The intensity of fluorescence was calculated using Image J (National Institutes of Health, USA).

### 2.12 Detection of the mitochondrial membrane potential

JC‐1 (5,5′,6,6′‐Tetrachloro‐1,1′,3,3′‐tetraethyl‐imidacarbocyanine iodide) was used to detect the mitochondrial membrane potential. In mitochondria with a normal and constant membrane potential difference, JC‐1 aggregates and fluoresces red, whereas when the membrane potential is decreased, JC‐1 exists as a monomer and fluoresces green. The PC12 cells were seeded in 24‐well plates and treated with 5‐PAHSA, S‐9‐PAHSA, and 9‐PAHSA for 24 h, then washed twice with a serum‐free medium. A JC‐1 dye work solution was prepared according to the instructions of the manufacturer (JC‐1 MitoMP Detection Kit, Dojindo Laboratories, Japan) and the PC12 cells were incubated in this solution for 45 min, followed by two washings in a serum‐free medium and observation under a fluorescence microscope.

### 2.13 Detection of GSH in the PC12 cells

In accordance with the instructions of the manufacturer (GSSG/GSH Quantification Kit II, Dojindo Laboratories, Japan), the samples or standards were added sequentially to a 96‐well plate, followed by the addition of 10 μL of the protein removal reagent M to the blank wells, standards of different concentrations to the standard wells, and samples to the sample wells. A 150 μL aliquot of total glutathione detection working solution was added to the wells and incubated at room temperature for 5 min, followed by the addition of 50 μL of NADPH solution and further incubation for 25 min at room temperature. The absorbance values were measured at 412 nm using a microplate spectrophotometer, with a standard curve prepared using the absorbance value of the standard wells. The GSH content in each sample was then calculated from the standard curve and the absorbance value in the sample well.

### 2.14 Statistical analysis

Measurement data with a normal distribution were expressed as mean ± standard error, while data with an abnormal distribution were expressed as the median and quartiles. The classification data were expressed as frequencies. The D'Agostino & Pearson test and Shapiro–Wilk test were used to test the normality of the data. Data with a normal distribution and homogeneity of variance were compared using the unpaired *t* test, while data with an abnormal distribution and homogeneity of variance were compared using the Mann–Whitney *U* test. One‐way analysis of variance (one‐way ANOVA) was used for the comparison between multiple groups with data with a normal distribution and homogeneity of variance, and the Kruskal–Wallis test for data with an abnormal distribution and homogeneity of variance. A *p* value of <0.05 was deemed to indicate statistical significance. All the analyses were conducted and the graphical representations were generated using GraphPad Prism 8.3 software (GraphPad Software, San Diego, USA).

## 3 RESULTS

### 3.1 S‐9‐PAHSA significantly decreased fasting blood glucose and serum LDL levels and increased insulin sensitivity in HFD mice

Fasting blood glucose was measured and an OGTT and ITT were performed to test the effect of S‐9‐PAHSA on glucose and lipid regulation. The results showed that S‐9‐PAHSA, but not 9‐PAHSA, significantly decreased FBG in the HFD mice in the third week (Figure 1B, *n* = 10–12 for each group, *p <* 0.001). Body weight did not change significantly in both the 9‐PAHSA and S‐9‐PAHSA groups (Figure 1C, *p >* 0.05). S‐9‐PAHSA also increased insulin sensitivity significantly in the HFD mice, but did not improve glucose tolerance or serum insulin levels. However, 9‐PAHSA did not improve insulin sensitivity, glucose tolerance, or serum insulin levels in the HFD mice (Figure 1D,F, *p <* 0.05 at 30 and 60 min in Figure 1E). Regarding blood lipids, LDL decreased significantly after administration of 9‐PAHSA and S‐9‐PAHSA (Figure 1G, *p <* 0.05), although there was no significant difference in TG levels between the 9‐PAHSA and S‐9‐PAHSA groups (Figure 1H, *p >* 0.05).

### 3.2 S‐9‐PAHSA did not significantly elevate cognition in HFD mice

**FIGURE 2:**
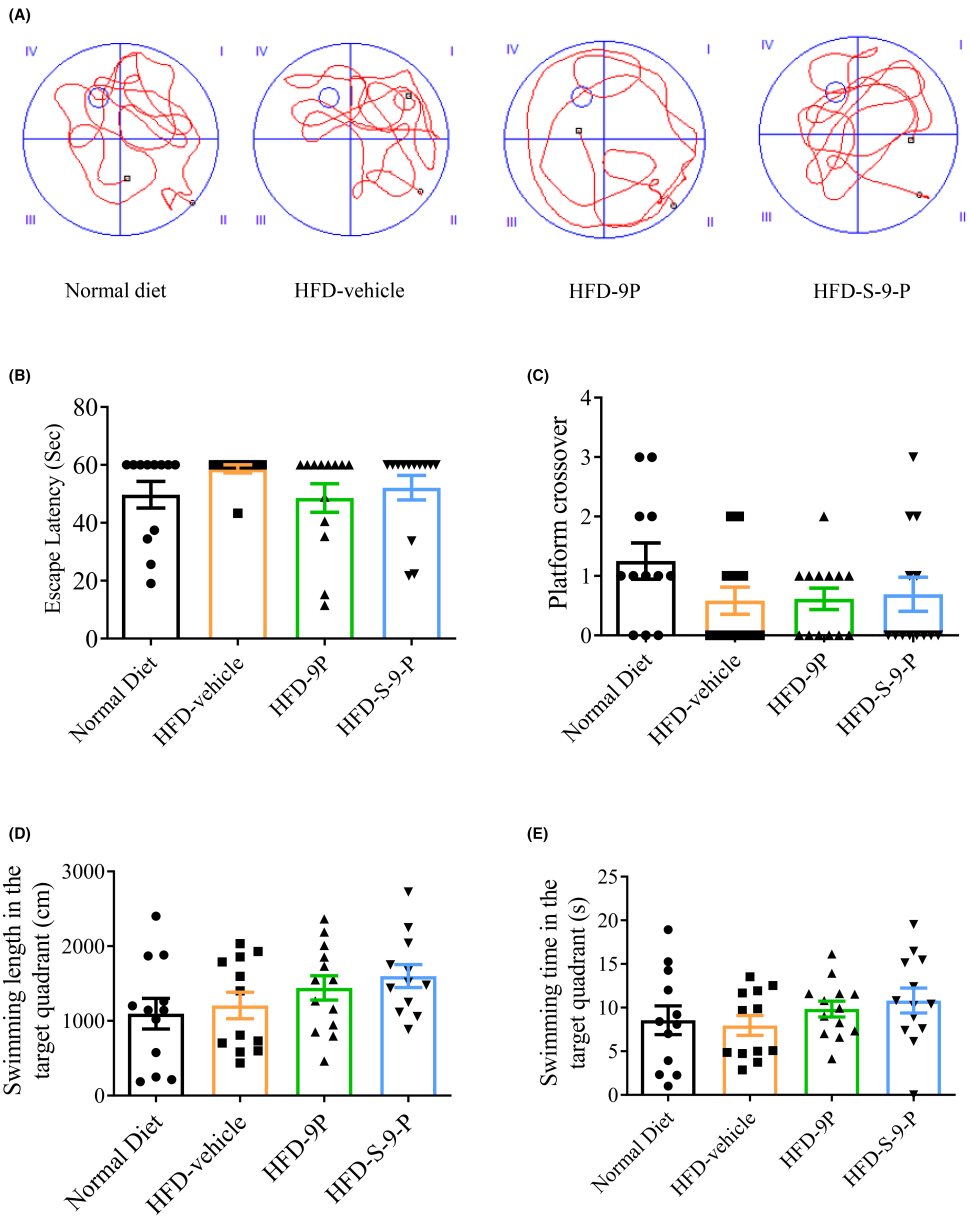
Behavior test of mice with different administrations for 4 weeks. (A) Representative trails of the mice in different groups. (B–E) Results of the Morris Water Maze test in the different groups (B, escape latency; C, times of platform crossover; D, swimming length in the target quadrant; E, swimming time in the target quadrant), *n* = 10–12. The data are shown as mean ± SEM. One‐way ANOVA was used in B–E.

The Morris water maze was used to test the potential effect of S‐9‐PAHSA to increase cognition. The representative trails in each group are shown in Figure 2A (*n* = 10–12 for each group). The results showed that compared with the vehicle group, the escape latency decreased slightly in the 9‐PAHSA and S‐9‐PAHSA groups. The times of platform crossover, swimming length, and time in target quadrants were elevated slightly in the 9‐PAHSA and S‐9‐PAHSA groups, although none of these results were statistically significant (Figure 2B–E, *p >* 0.05).

### 3.3 S‐9‐PAHSA significantly suppressed apoptosis and oxidative stress in PC12 cells and the cortex of mice under diabetic environments

**FIGURE 3:**
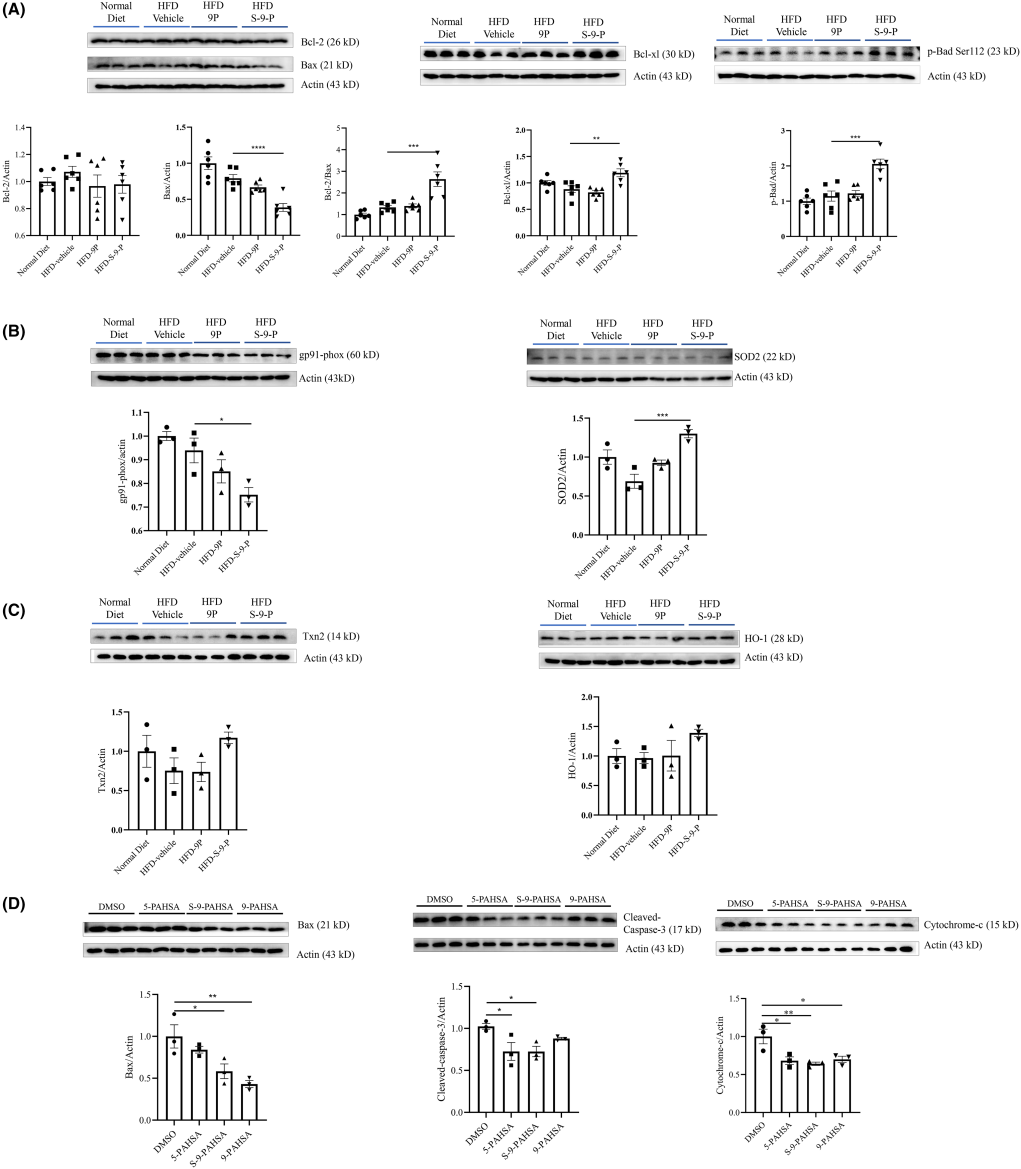
Expression of apoptosis‐ and oxidative stress‐related proteins after administration of S‐9‐PAHSA in mice and PC12 cells. (A) Expression of Bcl‐2, Bax, Bcl‐xl, and p‐Bad in the brain of mice receiving different administrations for 4 weeks, *n* = 6; (B–C) expression of oxidative stress‐related protein in the brain of mice receiving different administrations for 4 weeks. (B) gp91‐phox and SOD2; C, Txn2 and HO‐1, *n* = 3; (D) expression of Bax, cleaved‐caspase‐3, and cytochrome C in PC12 cells with different doses of PAHSA administration for 24 h, *n* = 3. *p < 0.05, **p < 0.01, ***p < 0.001, ****p < 0.0001. The data are shown as mean ± SEM. One‐way ANOVA was used in A–D.

To test the potential protective effects of S‐9‐PAHSA on neurons, the levels of apoptosis and oxidative stress‐related proteins were investigated in the HFD mice. The results showed that the apoptosis‐related protein, Bax was reduced significantly in the S‐9‐PAHSA group, and that the anti‐apoptosis proteins p‐Bad and Bcl‐xl, and the Bcl‐2/Bax ratio were increased significantly compared to those measured in the 9‐PAHSA group (Figure 3A, *n* = 6, *p <* 0.0001 for Bax, *p <* 0.001 for Bcl‐2/Bax, *p <* 0.01 for Bcl‐xl, *p <* 0.001 for p‐Bad). In terms of oxidative stress, the oxidative stress product gp91‐phox was decreased, while the antioxidant enzyme SOD2 was increased in the S‐9‐PAHSA group. In addition, the antioxidant proteins Txn2 and HO‐1 showed a slight increase, although this difference was not statistically significant (Figure 3B,C, *n* = 3, *p <* 0.05 for gp91‐phox, *p <* 0.001 in SOD2). In PC12 cells exposed to a diabetic environment, S‐9‐PAHSA demonstrated a greater ability to decrease levels of cleaved caspase‐3 and cytochrome‐C compared with that caused by 9‐PAHSA and 5‐PASHA (Figure 3D, *n* = 3, Bax: *p <* 0.05 for S‐9‐PAHSA, *p <* 0.01 for 9‐PAHSA; Cleaved caspase‐3: *p <* 0.05 for S‐9‐PAHSA and 9‐PAHSA; Cytochrome‐C: *p <* 0.05 for 5‐PAHSA and 9‐PAHSA, *p <* 0.01 for S‐9‐PAHSA).

### 3.4 CAIII played a key role in the protective effect of S‐9‐PAHSA under diabetic environments

**FIGURE 4:**
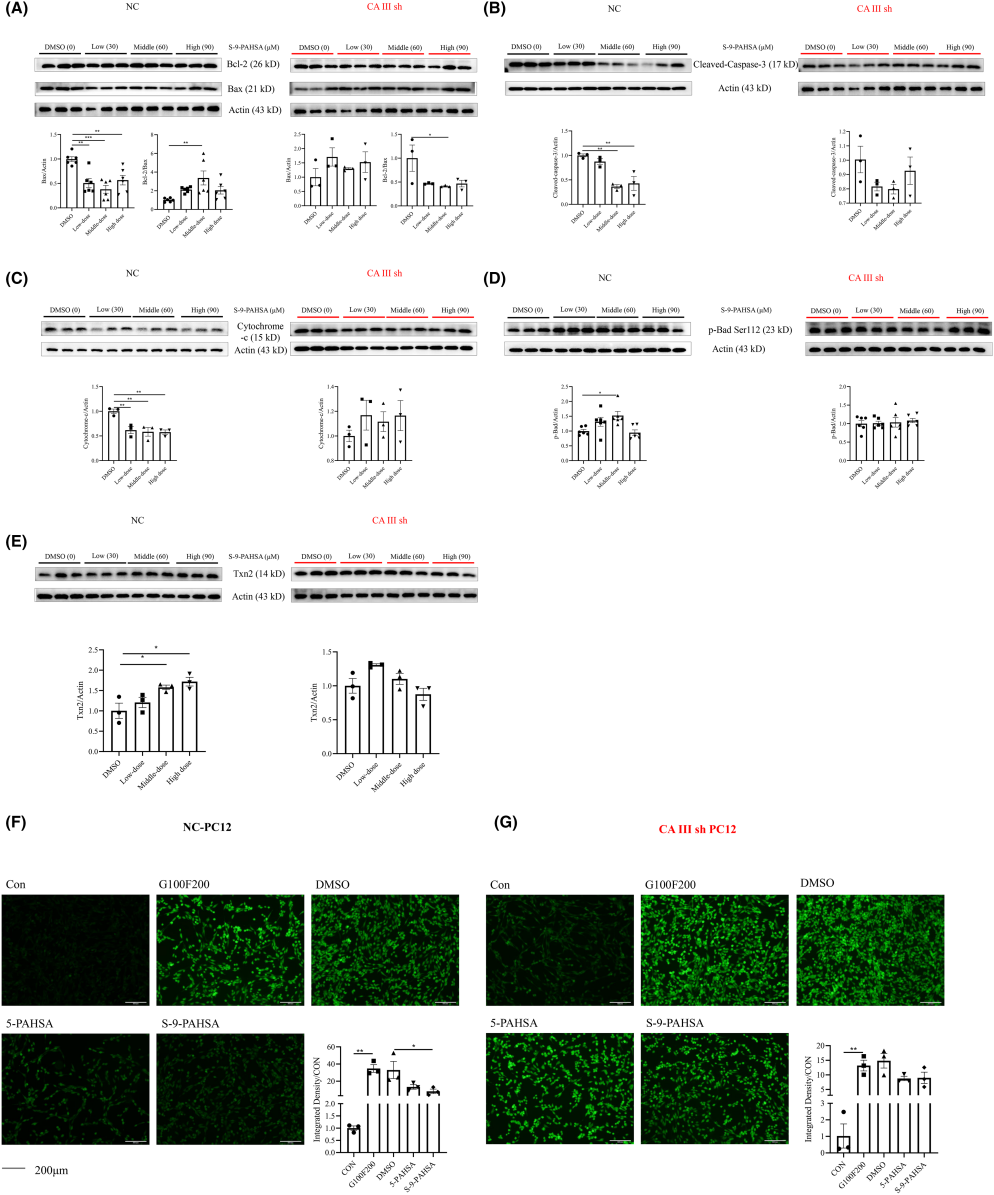
Levels of apoptosis‐ and oxidative stress‐related indicators in normal and CAIII knocked‐down PC12 cells after administration of S‐9‐PAHSA for 24 h. (A–D) Apoptosis‐related protein after administration of different concentrations of S‐9‐PAHSA in normal and CAIII knocked‐down PC12 cells (A, Bcl‐2/Bax; B, cleaved‐caspase‐3; C, cytochrome C; D, p‐Bad; *n* = 6 in A and D, *n* = 3 in B and C). (E) Levels of Txn2 in normal and CAIII knocked‐down PC12 cells after administration of S‐9‐PAHSA. F‐G, ROS in normal (F) and CAIII knocked‐down PC12 cells (G) after administration of 5‐PAHSA and S‐9‐PAHSA, *n* = 3. *p < 0.05, **p < 0.01, ***p < 0.001, ****p < 0.0001. The data are shown as mean ± SEM. Scale bar = 200 μm. Kruskal–Wallis test was used in A CAIII sh, D NC and E CAIII sh. One‐way ANOVA was used in other figures.

To further investigate the specific mechanism and key protein involved in the anti‐apoptosis and anti‐oxidative stress effects of S‐9‐PAHSA, we established the CAIII knocked‐down and overexpressed PC12 cell lines, respectively, and their corresponding controls. The results showed that in the vehicle groups, S‐9‐PAHSA was able to decrease apoptosis‐related proteins such as Bax and cleaved‐caspase‐3, while increasing the levels of the anti‐apoptosis indicators, p‐Bad and the Bcl‐2/Bax ratio. Of the three different dosages tested, the middle dosage (60 μM) showed the best protective effect. However, in the CAIII sh group, none of the three dosages had a protective effect on apoptosis‐related proteins under diabetic conditions (Figure 4A–D; Figure 4A, NC: Bax, *p <* 0.01 for 30 and 90 μM, *p <* 0.001 for 60 μM; Bcl‐2/Bax, *p <* 0.01 for 60 μM; Figure 4A, CAIII sh: Bcl‐2/Bax, *p <* 0.05 for 60 μM. Figure 4B, NC: Cleaved‐caspase‐3, *p <* 0.01 for 60 and 90 μM; Figure 4C, NC: Cytochrome C, *p <* 0.01 for 30, 60, and 90 μM; Figure 4D, NC: p‐Bad, *p <* 0.05 for 60 μM). Administration of S‐9‐PAHSA led to an elevation in the levels of anti‐oxidative stress proteins in the vehicle PC12 cells, although this effect was not observed in the CAIII sh PC12 cells (Figure 4E, NC: *p <* 0.05 for 60 and 90 μM). Our previous study had shown that 5‐PAHSA decreased the levels of ROS in PC12 cells under diabetic conditions and therefore, we used 5‐PAHSA as a control to compare the anti‐oxidative effect of S‐9‐PAHSA. Immunofluorescence analysis revealed that ROS levels were increased significantly under diabetic conditions in both the vehicle and CAIII sh PC12 cells. However, when different isomers of PAHSA were administered, the levels of ROS decreased in both the 5‐PAHSA and S‐9‐PAHSA groups in the vehicle PC12 cells, with the difference being more significant in the S‐9‐PAHSA group (Figure 4F, *n* = 3, *p <* 0.01 for G100F200, *p <* 0.05 in S‐9‐PAHSA). In the CAIII sh PC12 cells, no significant difference in ROS levels was observed in either the 5‐PAHSA or S‐9‐PAHSA groups (Figure 4G, *n* = 3, *p <* 0.01 for G100F200).

### 3.5 S‐9‐PAHSA restored mitochondrial function in PC12 cells under diabetic environments, with CAIII playing a key role in this process

**FIGURE 5:**
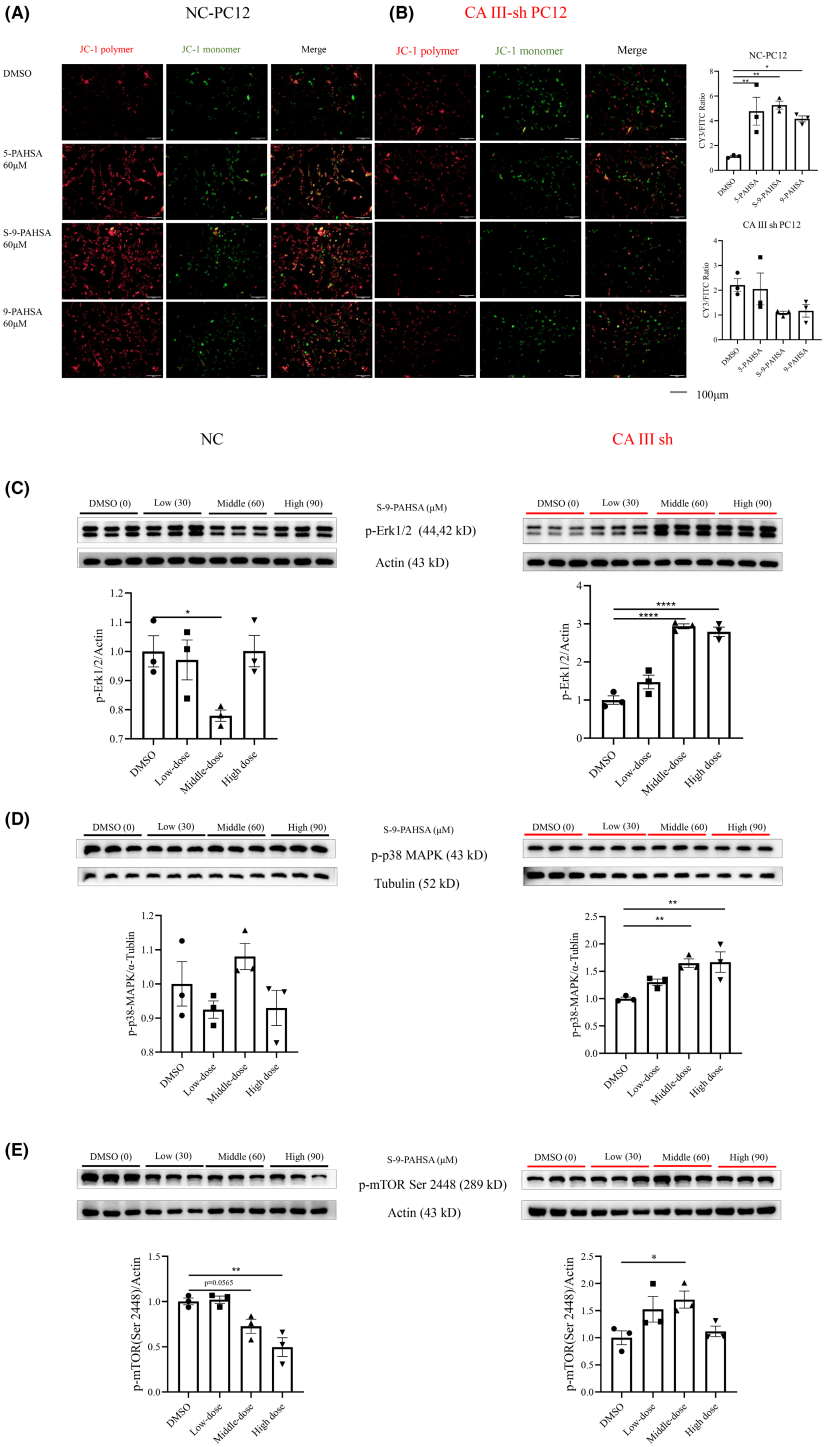
Mitochondrial function and m‐TOR/p38‐AMPK/Erk1/2 in normal and CAIII knocked‐down PC12 cells after administration of S‐9‐PAHSA for 24 h. (A, B) JC‐1 dye in normal (A) and CAIII knocked‐down PC12 cells (B) after administration of 5‐PAHSA, 9‐PAHSA, and S‐9‐PAHSA, *n* = 3, scale bar = 100 μm; (C–E) pro‐oxidative stress‐related proteins (C, p‐Erk1/2; D, p‐p38 MAPK; E, p‐TOR Ser2448, *n* = 3) in normal and CAIII knocked‐down PC12 cells after administration of different concentrations of S‐9‐PAHSA. *p < 0.05, **p < 0.01, ***p < 0.001, ****p < 0.0001. The data are shown as mean ± SEM. One‐way ANOVA was used in A–E.

Mitochondrial dysfunction is one of the typical pathologies associated with diabetic neuronal damage and can lead to apoptosis and disturbances in energy metabolism. In order to test the role of CAIII in the neuroprotective effect of PAHSA on mitochondrial function in a diabetic environment, we measured the mitochondrial membrane potential using JC‐1 dye. The results showed that in the vehicle groups, the CY3/FITC ratio was increased significantly in the 5‐PAHSA, S‐9‐PAHSA, and 9‐PAHSA groups, with the highest increase observed in the S‐9‐PAHSA group (Figure 5A, *n* = 3, *p <* 0.01 for 5‐PASHA and S‐9‐PAHSA, *p <* 0.05 for 9‐PAHSA). In the CAIII sh groups, the CY3/FITC ratio was decreased slightly in the S‐9‐PAHSA and 9‐PAHSA groups, although this difference was not statistically significant (Figure 5B, *n* = 3, *p >* 0.05).

### 3.6 Phosphorylation of m‐TOR/p38‐AMPK/Erk1/2 was enhanced significantly in CAIII knockdown PC12 cells after administration of S‐9‐PAHSA

Pro‐oxidative stress‐related proteins were detected using Western blotting to further investigate the possible mechanisms for weakening the protective effect of S‐9‐PAHSA after CAIII knockdown and also to evaluate the antioxidant effect of CAIII. The results showed that in CAIII sh PC12 cells, the phosphorylation of m‐TOR, p38‐MAPK, and Erk1/2 was enhanced significantly after administration of S‐9‐PAHSA, with the most significant trend observed at a concentration of 60 μM. In the vehicle PC12 cells, the phosphorylation of m‐TOR and Erk1/2 was suppressed significantly at concentrations of 90 and 60 μM (Figure 5C–E, *n* = 3, Figure 5C, NC: *p <* 0.05 at 60 μM; Figure 5C, CAIII sh, *p <* 0.0001 at 60 and 90 μM; Figure 5D, CAIII sh, *p <* 0.01 at 60 and 90 μM; Figure 5E, NC: *p <* 0.01 at 90 μM; Figure 5E, CAIII sh: *p <* 0.05 at 60 μM).

### 3.7 CAIII overexpression did not enhance the anti‐apoptosis and anti‐oxidative stress effects of S‐9‐PAHSA

**FIGURE 6:**
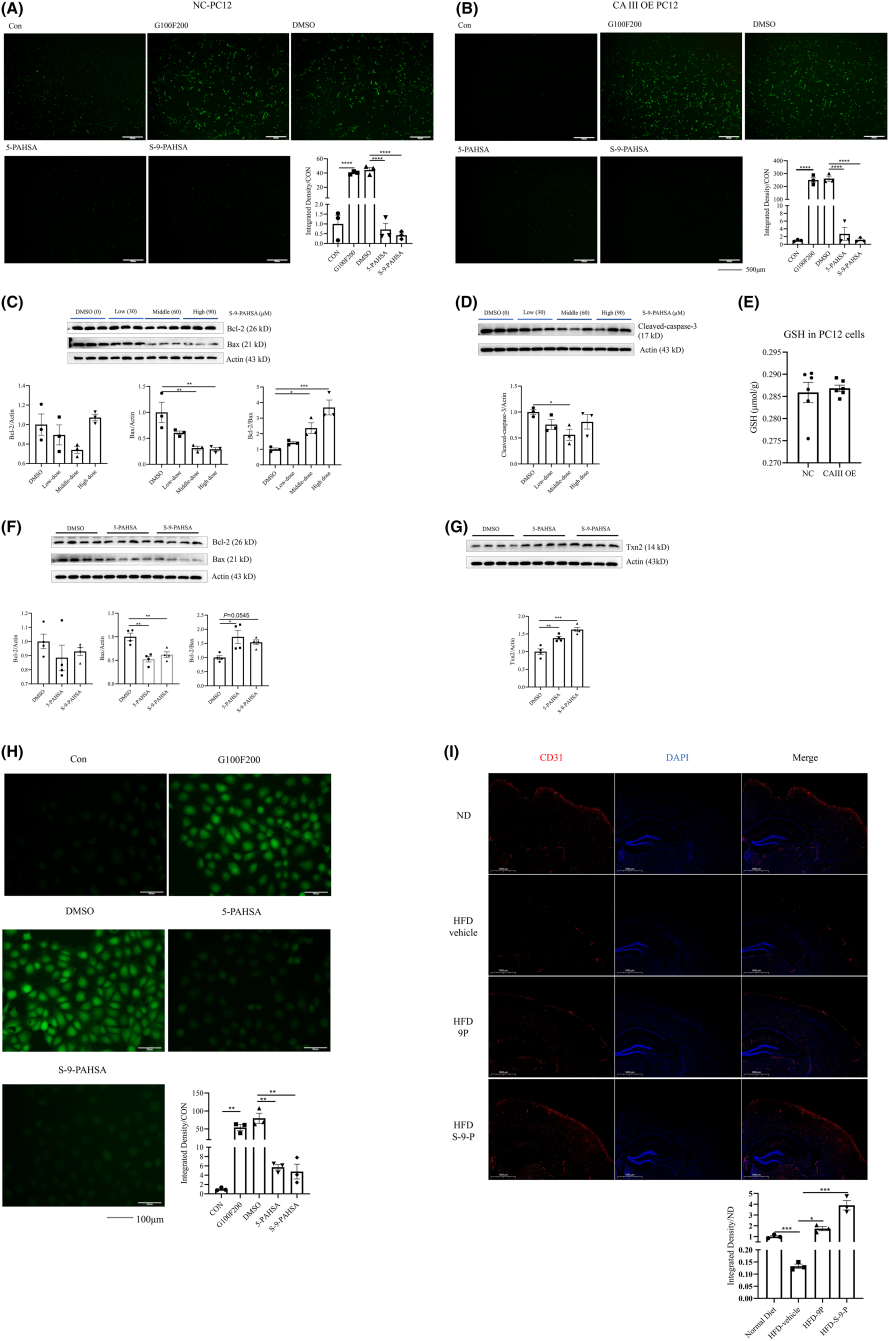
Levels of oxidative stress‐ and apoptosis‐related proteins in normal and CAIII overexpressed PC12 cells after administration of S‐9‐PAHSA for 24 h and the effect of S‐9‐PAHSA on endothelial cells for 24 h. (A, B) ROS in normal (A) and CAIII overexpressed PC12 cells; (B) after administration of 5‐PAHSA and S‐9‐PAHSA, *n* = 3, scale bar =500 μm. (C, D) Apoptosis‐related protein Bcl‐2, Bax (C), and cleaved‐caspase‐3 (D) in normal and CAIII overexpressed PC12 cells after administration of different concentrations of S‐9‐PAHSA, *n* = 4. (E) GSH in normal and CAIII overexpressed PC12 cells. (F) Bcl‐2 and Bax in RBE4 cells after administration of 5‐PAHSA and S‐9‐PAHSA, *n* = 6. (G) Txn2 in RBE4 cells after administration of 5‐PAHSA and S‐9‐PAHSA, *n* = 4. (H) ROS in RBE4 cells after administration of 5‐PAHSA and S‐9‐PAHSA, *n* = 3, scale bar = 100 μm. (I) CD31‐positive cells in mice administrated 9‐PAHSA and S‐9‐PAHSA for 4 weeks, *n* = 4, scale bar = 1000 μm. *p* < 0.05, **p < 0.01, ***p < 0.001, ****p < 0.0001. The data are shown as mean ± SEM. Kruskal–Wallis test was used in C Bcl‐2/Bax, unpaired *t*‐test was used in E. One‐way ANOVA was used in other figures.

The above experiments showed the important role CAIII has in the protective effect of S‐9‐PAHSA under diabetic conditions. We, therefore, established CAIII overexpressed (OE) PC12 cells to investigate whether the protective effect of S‐9‐PAHSA could be enhanced under diabetic conditions. Immunofluorescence staining showed that S‐9‐PAHSA significantly decreased ROS in both vehicle and CAIII OE PC12 cells, with this effect being more pronounced in the cells overexpressing CAIII (Figure 6A,B, *n* = 3, *p <* 0.0001 in the G100F200, 5‐PAHSA, and S‐9‐PAHSA groups). In addition, S‐9‐PAHSA decreased expression of the apoptosis‐related proteins Bax and cleaved‐caspase‐3 in CAIII OE PC12 cells, although this effect was not significantly better than that observed in the vehicle PC12 cells (Figure 6C,D, *n* = 3, Bax, *p <* 0.01 for 60 and 90 μM; Bcl‐2/Bax, *p <* 0.001 for 90 μM; cleaved caspase‐3: *p <* 0.05 for 60 μM). Previous studies have reported that the anti‐oxidative stress effect of CAIII was dependent on sufficient GSH.^37^ Therefore, we measured the levels of GSH in both vehicle and CAIII overexpressed PC12 cells and showed no significant difference between the two cell lines (Figure 6E, *n* = 6, *p >* 0.05).

### 3.8 S‐9‐PAHSA protected endothelial cells from apoptosis and oxidative stress under diabetic environments

Microvascular dysfunction has been identified as another factor that contributes to diabetes‐related neurodegeneration. Given the neuroprotective effects of S‐9‐PAHSA on neuronal cells in a diabetic environment, we also investigated its potential in vitro for microvascular protection, using 5‐PAHSA as the control. S‐9‐PAHSA significantly decreased expression of the apoptosis‐related protein Bax and increased anti‐oxidative stress protein Txn2, with this effect superior to that observed with 5‐PAHSA (Figure 6F,G, *n* = 4; Bax, *p <* 0.05 for 5‐PAHSA; Bcl‐2/Bax, *p <* 0.05 for 5‐PAHSA and *p <* 0.01 for S‐9‐PAHSA; Txn2, *p <* 0.01 for 5‐PAHSA and *p <* 0.001 for S‐9‐PAHSA). Immunofluorescence staining showed that ROS was increased significantly in RBE4 cells under diabetic environments and that both 5‐PAHSA and S‐9‐PAHSA had the ability to significantly decrease ROS levels in PC12 cells under diabetic conditions, with S‐9‐PAHSA exhibiting superior efficacy (Figure 6H, *n* = 3; *p <* 0.001 for G100F200, 5‐PAHSA, and S‐9‐PAHSA groups). In the in‐vivo study, the density of CD31‐positive cells in the cortex of the HFD mice was decreased significantly compared to that in the chow‐fed mice. However, in the 9‐PAHSA and S‐9‐PAHSA groups, the density of CD31‐positive cells was increased significantly, with a more significant increase observed in the S‐9‐PAHSA group (Figure 6I, *n* = 3; *p <* 0.001 for HFD, *p <* 0.05 for 9‐PAHSA, and *p <* 0.001 for S‐9‐PAHSA). These findings demonstrated that S‐9‐PAHSA had the ability to protect cerebrovascular endothelial cells in a diabetic environment by suppressing apoptosis and oxidative stress.

## 4 DISCUSSION

The incidence and disability rate of neurological complications caused by T2DM in the elderly is increasing every year.^5^ Diabetes‐induced insulin resistance and vascular damage result in cerebral hypoperfusion, ischemia, and hypoxia, further exacerbating neurodegeneration and cognitive dysfunction. When neuronal and vascular complications coexist, they create a vicious cycle in which the two pathologies interact with each other.^38^ Therefore, addressing and identifying interventions and therapeutic targets for diabetic neuronal and vascular complications remain a crucial aspect of both basic and clinical research.

Current studies on PAHSA have focused mainly on whether it ameliorates insulin resistance and protects islet β cells, thereby promoting glucose transport.^16^, ^17^, ^18^ However, the effect of PAHSA on diabetic neurodegeneration remains unclear. Our previous studies have shown that 9‐PAHSA protects cardiac function and cardiomyocytes by enhancing autophagy flux. Likewise, 5‐PAHSA protects neuronal cells in diabetic environments by enhancing autophagy and inhibiting oxidative stress, although it does not suppress apoptosis in these cells.^19^, ^32^ Furthermore, there is evidence that 5‐PAHSA is unable to reduce fasting blood glucose levels or improve cognitive decline.^32^ 9‐PAHSA has been reported to have two distinct racemic isomers, S‐9‐PAHSA and R‐9‐PAHSA. Due to the S‐9‐PAHSA isomer stimulating insulin secretion and glucose intake to a greater extent than R‐9‐PAHSA,^22^ we used it as the main intervention in the studies described in this paper.

Our investigations on S‐9‐PAHSA had the aim of illustrating its possible protective effect on neurons in a mouse model of T2DM. FBG levels decreased significantly in the third week after S‐9‐PAHSA administration, but not following the administration of 9‐PAHSA. This suggests that S‐9‐PAHSA is more effective for decreasing FBG levels in HFD mice than 9‐PAHSA, possibly due to the racemic structure of S‐9‐PAHSA. This result indicated that further investigation of PAHSA's specific effect of decreasing FBG was warranted. ITT showed blood glucose levels were decreased significantly 30 and 60 min after insulin injection in the S‐9‐PAHSA group, indicating that S‐9‐PAHSA, but not 9‐PAHSA, had the ability to enhance insulin sensitivity in HFD mice. In addition, neither S‐9‐PAHSA nor 9‐PAHSA decreased blood glucose levels in the HFD mice during an OGTT. This result while consistent with Syed's research which showed that S‐9‐PAHSA did not improve glucose tolerance in HFD mice, differed from the results of other previous reports on PAHSA.^17^, ^18^ The average age of mice in our study at the time the ITT and OGTT were conducted was 44 weeks, which was markedly different from the ages in these two previous studies, which were 15 and 20–30 weeks, respectively. This variation in the age of the mice may explain the differences we observed as there is evidence that aging may contribute to impaired glucose tolerance.^39^

The current study showed that serum LDL, but not TG, was increased significantly in HFD mice compared with levels measured in mice fed a chow diet. These findings are consistent with previous studies on lipid metabolism in HFD mice.^40^ Our experiments also showed that both S‐9‐PAHSA and 9‐PAHSA significantly decreased LDL levels in HFD mice, but had no significant effect on serum TG. These results suggest that S‐9‐PAHSA has the potential to decrease LDL in T2DM.

Current studies on PAHSA have focused mainly on metabolic‐related organs such as the liver, islet cells, and adipose tissue, there have been relatively few studies on its neuroprotective effects. The current study showed that administration of S‐9‐PAHSA and 9‐PAHSA was associated with slight improvements in cognitive function in HFD mice, although this difference was not statistically significant. This result could be attributed to the following changes we observed. First, the cognition of mice fed a normal diet also decreased, with the animals showing no significant difference in escape latency in the Morris water maze compared to that of the HFD mice. The T2DM mice used in our study were 11 months old when the behavioral tests were conducted, and combined with a 6‐month HFD, these two physiological and pathological factors led to a significant baseline decline in cognition. This decline may have partially obscured the effectiveness of S‐9‐PAHSA in improving cognitive function. Moreover, the Morris water maze experiment stipulates a maximum latency of 60 s,^41^ with the average latency observed in the HFD group being 58.61 seconds, a duration which nearly approached the maximum time. This result may explain why no significant difference was observed between the vehicle and S‐9‐PAHSA/9‐PAHSA groups. Our previous study indicated that 9‐PAHSA improved the cognition of DB/DB mice. However, in this study, neither S‐9‐PAHSA nor 9‐PAHSA had a significant effect on the cognition of the HFD mice. The difference in these two studies may be attributable to the use of different animal models and experiments to test cognition.^21^

Given the trend of improved cognition we observed in the HFD mice, we also investigated the effect of S‐9‐PAHSA on anti‐apoptosis. The results showed that S‐9‐PAHSA had a significant impact by reducing apoptosis‐related proteins in the cortex of HFD mice in vivo and in neuronal cells under diabetic conditions in vitro. This suggests that S‐9‐PAHSA has the ability to inhibit apoptosis in neuronal cells. In addition, it is worth noticing that the expression of apoptosis‐related proteins in the cortex of HFD mice was similar to that of mice on a chow diet, and that previous studies had also reported a significant increase in the levels of apoptosis protein in the cortex of a D‐galactose‐induced aged mouse model.^42^ Therefore, these results may be attributed to the age of the animals in the various mouse models. Interestingly, the level of apoptosis‐related protein in the cortex of mice fed an HFD following S‐9‐PAHSA administration was significantly lower than that observed in the group fed a chow diet. This suggests that S‐9‐PAHSA may potentially improve aging‐related apoptosis, thereby providing a theoretical basis for its effect on neuronal apoptosis related to aging, similar to that reported in previous studies.^43^, ^44^, ^45^

Excessive oxidative stress is another mechanism that contributes to neuronal pathology in diabetes.^46^ Many studies have proven that suppressing oxidative stress alleviates neuronal damage in patients with diabetes.^15^, ^47^ For example, NAPDH oxidase‐like gp91‐phox was reported to induce the generation of oxidative radicals and play a key role in damage to the central nervous system,^48^ while L‐3‐n‐butylphthalide was shown to suppress oxidative stress by increasing SOD2 and downregulating gp91‐phox, thereby modulating synaptic plasticity and improving cognition.^49^ Txn2 and HO‐1 are also key proteins in anti‐oxidative stress and play a neuroprotective role.^50^ In the current study, the expression of gp91‐phox was decreased significantly and SOD2 was increased significantly in the cortex of HFD mice after S‐9‐PAHSA administration. Txn2 and HO‐1 also showed an increasing tendency, although the difference observed was not statistically significant. These results suggest that S‐9‐PAHSA has the potential to alleviate neuronal damage by suppressing oxidative stress in the cortex of HFD mice.

As an enzyme with antioxidant effects, CAIII has been shown to have several similarities to S‐9‐PAHSA, such as an ability to combat oxidative stress and a relationship with diabetes.^29^, ^51^ CAIII plays a crucial role in reducing oxidative stress and also appears to be essential for cognition, with evidence that it is downregulated in the cortex and myocardium of diabetic mouse models.^27^, ^29^ Based on these results, we speculated that CAIII may be involved in the anti‐oxidative stress and anti‐apoptotic effects of S‐9‐PAHSA. Therefore, we established CAIII knockdown and overexpressed cell lines to investigate the influence of CAIII on the protective effect of S‐9‐PAHSAs under diabetic conditions. The results showed that S‐9‐PAHSA's anti‐apoptotic effect was decreased significantly in CAIII knockdown PC12 cells under diabetic conditions, whereas the effect remained in vehicle PC12 cells. The effect was most significant at a concentration of 60 μM. This finding further confirmed that S‐9‐PAHSA's protective effect was associated with its anti‐apoptosis role. These apoptosis‐related proteins are all related to mitochondrial function, and therefore it is possible that the protective effect of S‐9‐PAHSA might be related to the maintenance of mitochondrial function.^52^, ^53^, ^54^, ^55^

Our previous in vitro study showed that 5‐PAHSA significantly decreased ROS but did not decrease apoptosis‐related proteins in PC12 cells under diabetic environments.^32^ These experiments demonstrated that S‐9‐PAHSA decreased intracellular ROS in PC12 cells under diabetic environments and that this effect was greater than that observed with 5‐PAHSA. This result may therefore explain why S‐9‐PAHSA has a greater suppressive effect on apoptosis compared to that of 5‐PAHSA. Furthermore, S‐9‐PAHSA did not decrease ROS in CAIII knockdown PC12 cells under diabetic environments, proving that CAIII also plays a key role in anti‐oxidative stress. These results confirmed that S‐9‐PAHSA has an anti‐oxidant effect under diabetic conditions and that CAIII has an essential role in the mechanism of this effect.

Apoptosis proteins such as Bax which were decreased by S‐9‐PAHSA are related to mitochondrial function, with oxidative stress also known to be one of the main causes of mitochondria dysfunction.^56^ The results showed that administration of the three types of PAHSA all resulted in an increase in mitochondrial membrane potential in the vehicle PC12 cells but not in the CAIII knockdown PC12 cells. This suggested that PAHSA alleviated mitochondrial dysfunction under diabetic environments and that CAIII was essential in this process. We also showed that S‐9‐PAHSA had a superior protective effect than 9‐PAHSA and 5‐PAHSA, a finding that corresponded to the results of our apoptosis‐related and anti‐oxidative stress in‐vitro studies.

The results also showed that S‐9‐PAHSA increased phosphorylation of Erk1/2 and p‐38 MAPK, common pathways involved in the activation of oxidative stress. There is evidence that platelet‐activating factors induce the expression of Erk1/2 and p38‐MAPK, leading to oxidative stress damage, while p38‐MAPK inhibitors and other oxidative stress molecules mitigate this damage.^57^ In addition, it has been reported that the Erk1/2 inhibitor, PD98059 exerts oxidative stress and anti‐apoptotic effects by inhibiting activation of Erk1/2 and caspase‐3, thereby alleviating brain damage in rats with cardiac arrest.^58^ Combined with the experimental results for the NC‐PC12 cells, we showed that 60 μM of S‐9‐PAHSA had a significant inhibitory effect on Erk1/2 activation, a finding that was consistent with the optimal concentration for S‐9‐PAHSA's anti‐oxidative stress and anti‐apoptosis properties, and also confirming the role of CAIII as a key molecule in the anti‐oxidant effect of S‐9‐PAHSA.

We also constructed PC12 cell lines that overexpressed CAIII to investigate whether excessive CAIII enhanced the protective effect of S‐9‐PAHSA. S‐9‐PAHSA did not show greater anti‐apoptosis and anti‐oxidative stress effects in these cells. A previous study in rat hepatocytes reported that sufficient GSH was necessary for CAIII's biological activity and that when GSH was removed by diethyl maleate and menadione was used to induce oxidative stress, irreversible oxidative stress damage occurred.^59^ Our results showed that there was no significant difference in GSH levels between vehicle and CAIII overexpressed PC12 cells, which may explain why S‐9‐PAHSA did not cause a superior effect in CAIII overexpressed PC12 cells.

In neurodegenerative diseases, microvascular dysfunction of the brain often precedes pathological changes in the central nervous system.^60^ CD31 is often used as a marker in endothelial cells to reflect microvascular density and function. In addition, a metalloprotease‐mediated decline in CD31 levels in diabetic settings has been reported to lead to retinal vascular microenvironment abnormalities.^61^, ^62^ In HFD mice, we observed that the density of CD31‐positive cells decreased significantly, suggesting that the density of the microvascular was also reduced. After administration of S‐9‐PAHSA and 9‐PAHSA, the density of CD31‐positive cells increased significantly, with this trend more significant in the S‐9‐PAHSA group. Our in vitro study also showed that S‐9‐PAHSA decreased apoptosis and oxidative stress‐related proteins and reduced intracellular ROS in RBE4 cells, with these effects being greater than those caused by 5‐PAHSA.

This study had several advantages. First, the novel type of PAHSA, S‐9‐PAHSA was proven to have a neuroprotective effect by suppressing apoptosis and oxidative stress in both the cortex of a T2DM mouse model and neuronal cells. These effects have not been reported previously. In addition, S‐9‐PAHSA decreased fasting blood glucose levels and improved insulin sensitivity in the HFD‐induced T2DM mouse model, which further proved its potential beneficial role in glucose metabolism. Finally, CAIII was identified as a key protein in the neuroprotective effects of S‐9‐PAHSA and was involved in anti‐oxidative stress and apoptosis.

Several weaknesses remain in this study. Due to limitations in technology, differences in the concentration of S‐9‐PAHSA in the brain and serum between the mice fed either an HFD or a chow diet could not be detected. Downregulation and overexpression of CAIII were also only conducted in the in‐vitro experiments but not in the in‐vivo experiments. The specific mechanisms of S‐9‐PAHSA's effect on increasing insulin sensitivity were also not determined. Therefore, further studies need to be conducted to clarify these issues and reveal more specific mechanisms involved in the neuroprotective effects of S‐9‐PAHSA.

In conclusion, this study showed that S‐9‐PAHSA has a neuroprotective effect by suppressing apoptosis and oxidative stress and protecting mitochondrial function, and that CAIII has important roles in this mechanism of action.

## FUNDING INFORMATION

This work was supported by the National Key R&D Plan “Intergovernmental International Science and Technology Innovation Cooperation” Key Special Project (2021YFE0111800), National Scientific Foundation of China (81871098 to Hou‐guang Zhou and 81671392 to Jing‐chun Guo), Three Year Action Plan for the Innovation and Development of Traditional Chinese Medicine in Shanghai (ZY(2021‐2023)‐0207‐01), and Shanghai Municipal Key Clinical Specialty (Geriatrics, No. shslczdzk02802).

## CONFLICT OF INTEREST STATEMENT

The authors state no potential conflict of interest for the research, authorship, and publication of this article.

## ETHICS STATEMENT

This study was proved by the Animal Welfare and Ethics Group, Department of Laboratory Animal Science, Fudan University (2020‐Huashan hospital‐JS190).

## Supporting information


Appendix S1

Appendix S2

## Data Availability

The data that support the findings of this study are available in the supplementary material of this article.
