# Supplementary figures and images for: S‐9‐PAHSA's neuroprotective effect mediated by CAIII suppresses apoptosis and oxidative stress in a mouse model of type 2 diabetes
Source: CNS Neurosci Ther. 2024 Feb 8;30(2):e14594. doi: 10.1111/cns.14594 (PMC10853598; doi:10.1111/cns.14594)

**The synthesis of S-9-PAHSA**

1H NMR


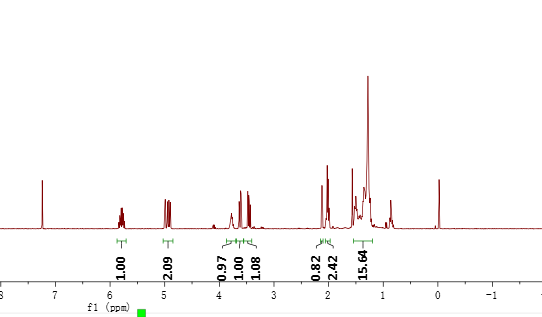


1H NMR


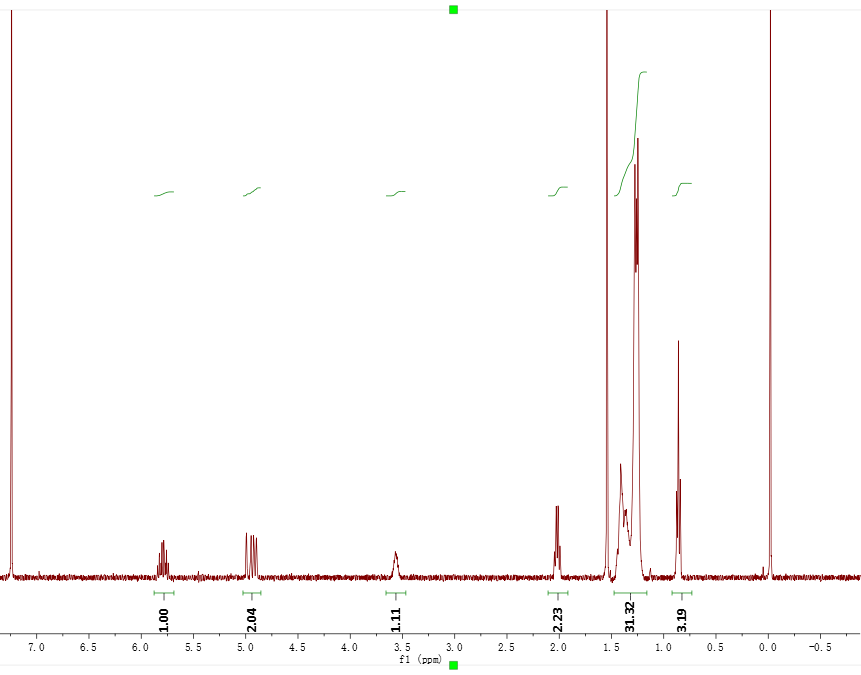


1H NMR


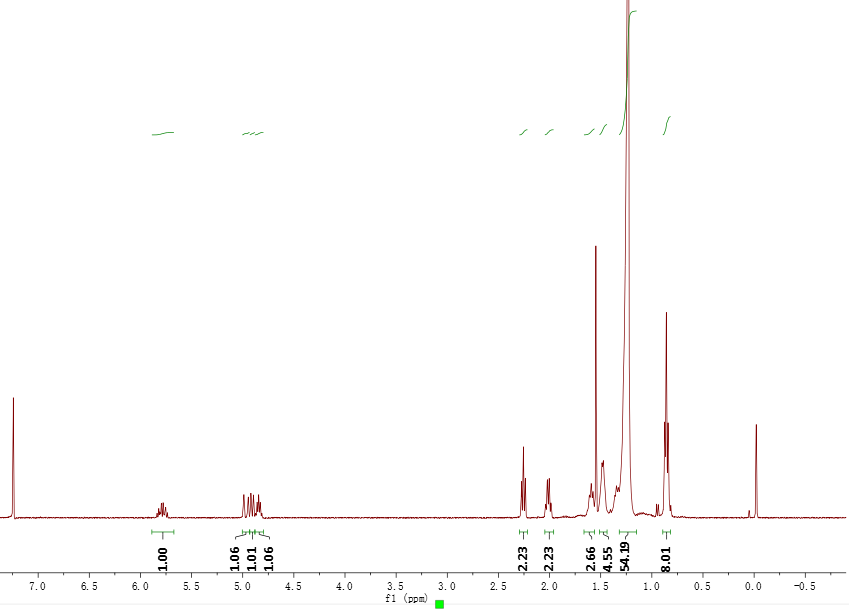


1H NMR

**(S-9-PAHSA)**


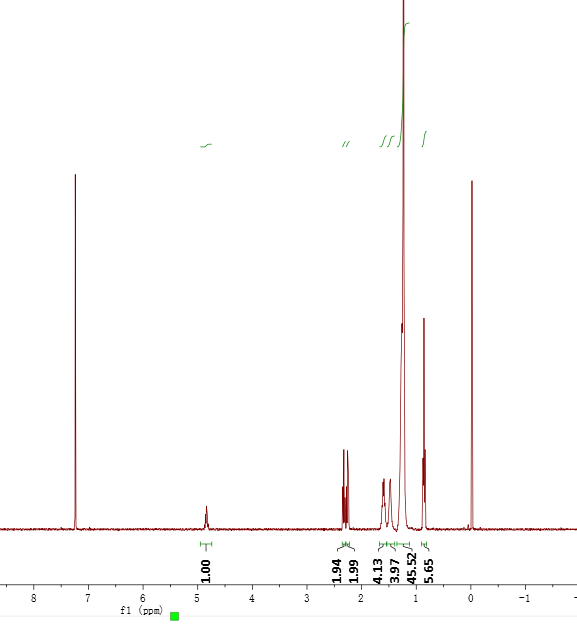

Supplement: Supplementary file 2 — Appendix S2 [file CNS-30-e14594-s002.docx]
